# Laryngeal sarcomatoid carcinoma: a case report and literature review on potential molecular targets for therapeutic opportunities

**DOI:** 10.3389/fonc.2025.1549790

**Published:** 2025-03-31

**Authors:** Jie Fan, Lun Chen, Chen-Hui Li, Zhong-Yong Xiao, Shui-Hong Zhou

**Affiliations:** ^1^ Department of Otolaryngology, Head and Neck Surgery, Ningbo No.9 Hospital, Ningbo, China; ^2^ Department of Otolaryngology, Head and Neck Surgery, The First Affiliated Hospital of Medical College of Zhejiang University, Hangzhou, China

**Keywords:** laryngeal sarcomatoid carcinoma, case report, molecular targets, therapeutical target, molecular markers

## Abstract

Laryngeal sarcomatoid carcinoma (LSC) is a rare variant of laryngeal malignancies characterized by an aggressive nature and poor prognosis, predominantly affecting older males. Although early diagnosis may facilitate organ preservation through adjuvant chemotherapy and radiation therapy, advanced stages of the disease, as classified by the TNM system, necessitate a deeper understanding of molecular interactions. This understanding could potentially yield improved molecularly targeted therapeutic opportunities and early diagnosis that likely support the treatment benefits in the LSC. Therefore, this study aims to identify possible molecular targets in LSC to better inform therapeutic options and prognostic markers for obtaining treatment benefits, alongside presenting a case study of a patient with LSC who was admitted to our department.

## Introduction

Laryngeal sarcomatoid carcinoma (LSC) is a rare type of laryngeal malignancy that tends to have a poor prognosis, even when detected at early stages. It is considered a more aggressive variant of squamous cell carcinoma of the head and neck. This condition typically affects older males. The World Health Organization’s 2017 Classification of Head and Neck Tumors states that LSC is a monoclonal neoplasm that originates from a non-committed stem cell, resulting in the development of both epithelial and mesenchymal components (1). Research indicates that the LSC defines the occurrence of epithelial-mesenchymal transition (2) and is recognized as a biphasic tumor consisting of two components: a squamous cell carcinoma and a malignant spindle cell component that exhibits a mesenchymal phenotype (2). This malignancy originates from an epithelial cell clone that has undergone mutations. LSC is a rare form of malignant variant that comprises 2 to 3% of all laryngeal cancers (3). As mentioned, this type onsets more specifically in elderly patients with a significant smoking history. Treatment options such as adjuvant chemotherapy and adjuvant radiation therapy can preserve the organ to treat the selected laryngeal cancers, which opens a new door in the treatment of LSC. In addition, suspension laryngoscopy and CO2 laser resection have produced a significant impact on this field, especially in treating the early stages of larynx cancers (4). Radiation therapy has also been remarkably improved over the decades. However, the incidence of LSC has been decreasing in recent decades, with an increase in mortality. This may be due to the poor prognosis, suggesting that identifying possible molecular biomarkers as prognostic markers or therapeutical targets can significantly improve the treatment outcomes in the LSC. Therefore, this review aims to find molecular markers as potential prognostic markers and therapeutic targets for the LSC, along with presenting a case report. For that, we reported a 68-year-old male patient with laryngeal sarcomatoid carcinoma admitted to our department in addition to presenting relevant literature for the aim of understanding possible molecular therapeutical strategies for LSC.

## Clinical case report

This case report presents a 68-year-old male patient diagnosed with LSC who was admitted to our department. The patient reported experiencing recurrent hoarseness over the past one year. He had a history of type 2 diabetes mellitus (T2DM), smoking habits for more than 30 years, and a history of alcohol consumption for the same duration. The onset of hoarseness occurred without an identifiable cause approximately one year prior and had progressively deteriorated since then. The patient encountered difficulties with vocalization, leading to a complete loss of voice; however, he had no difficulty in breathing or swallowing. Following treatment with anti-infective and anti-inflammatory therapies, there was an improvement in his hoarseness, but it was prone to recurrence. The patient’s hoarseness persisted for one year, and it gradually intensified over the year.

### Laryngoscopy

**Figure 1:**
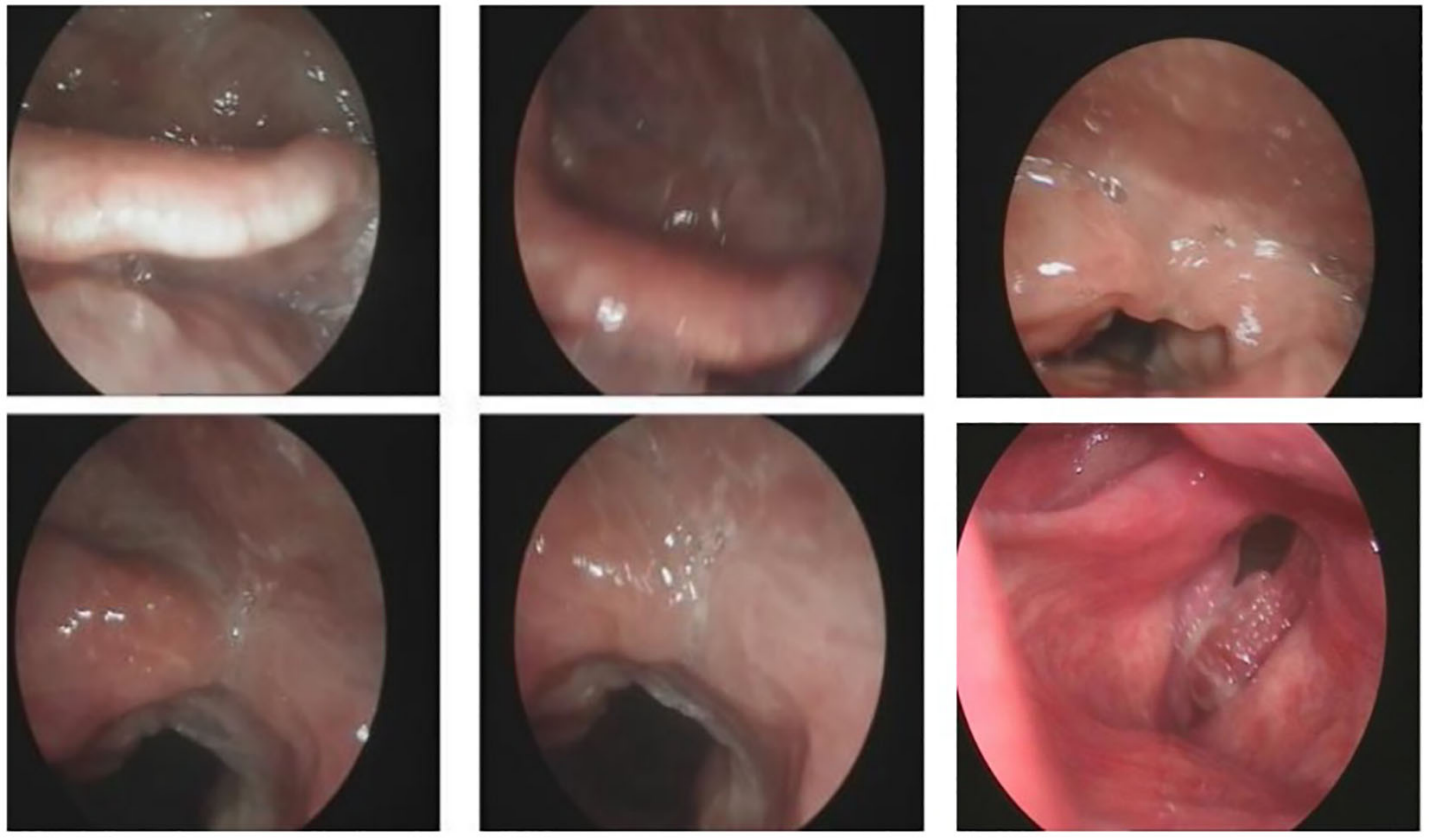
Laryngoscopy biopsy shows a malignant tumor with poor differentiation and necrosis (left vocal cord lesion, biopsy).

**Figure 2:**
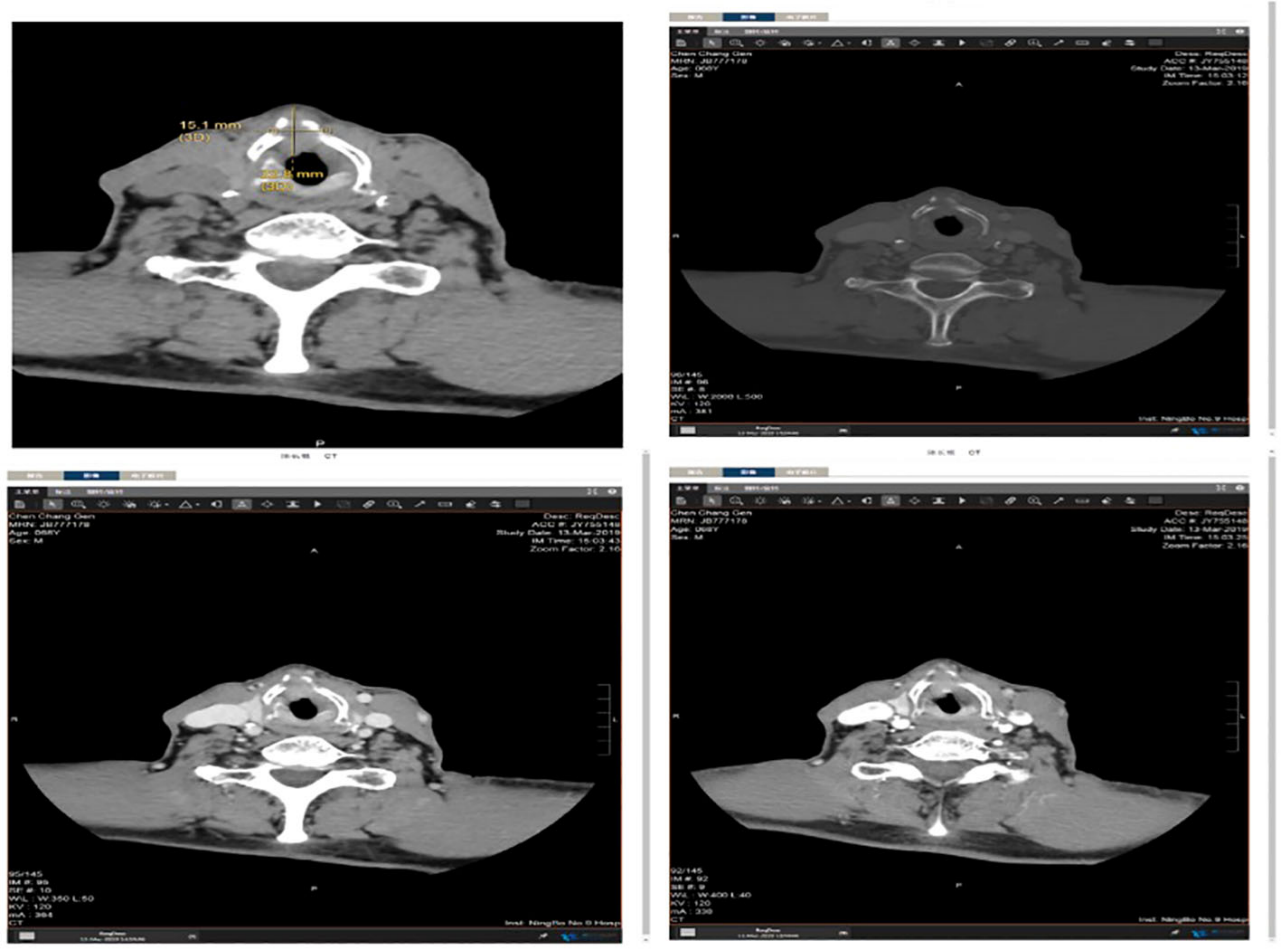
Larynx tomography (CT) scan showing that the morphology of the right vocal cord is fixed with thickening, about 6mm, extending forward to the anterior commissure of the vocal cord and the anterior edge of the left vocal cord.

Local hyperplasia of the left vocal cord, cumulative anterior commissure, and rough surface were observed (**Figure 1**). Larynx tomography (CT) scan shows that the morphology of the right vocal cord was fixed with thickening, about 6mm, extending forward to the anterior commissure of the vocal cord and the anterior edge of the left vocal cord (**Figure 2**). After enhancement, there was significant enhancement in the arterial phase, and the enhancement in the venous phase was higher than in the surrounding tissue. The bone window shows local bone destruction of the thyroid cartilage. No signs of mass were observed in the posterior space of the ring. The soft tissue structure of the remaining neck was symmetrical, and there was no apparent mass or enlarged lymph node shadow. The trachea was centered, the thyroid gland was not enlarged, and the density was symmetrical and uniform on both sides. The laryngeal cavity shown was unobstructed, with epiglottic valleys and symmetrical on both sides. The standard laryngeal carcinoma classification is based on tumor size, lymph node affection, and metastasis (TNM). It is the classification scheme of the American Joint Committee on Cancer Staging (AJCC), and it is used in the same way for stage spindle cell carcinoma (SPCC).

### Tumor classification of the patient

**Figure 3:**
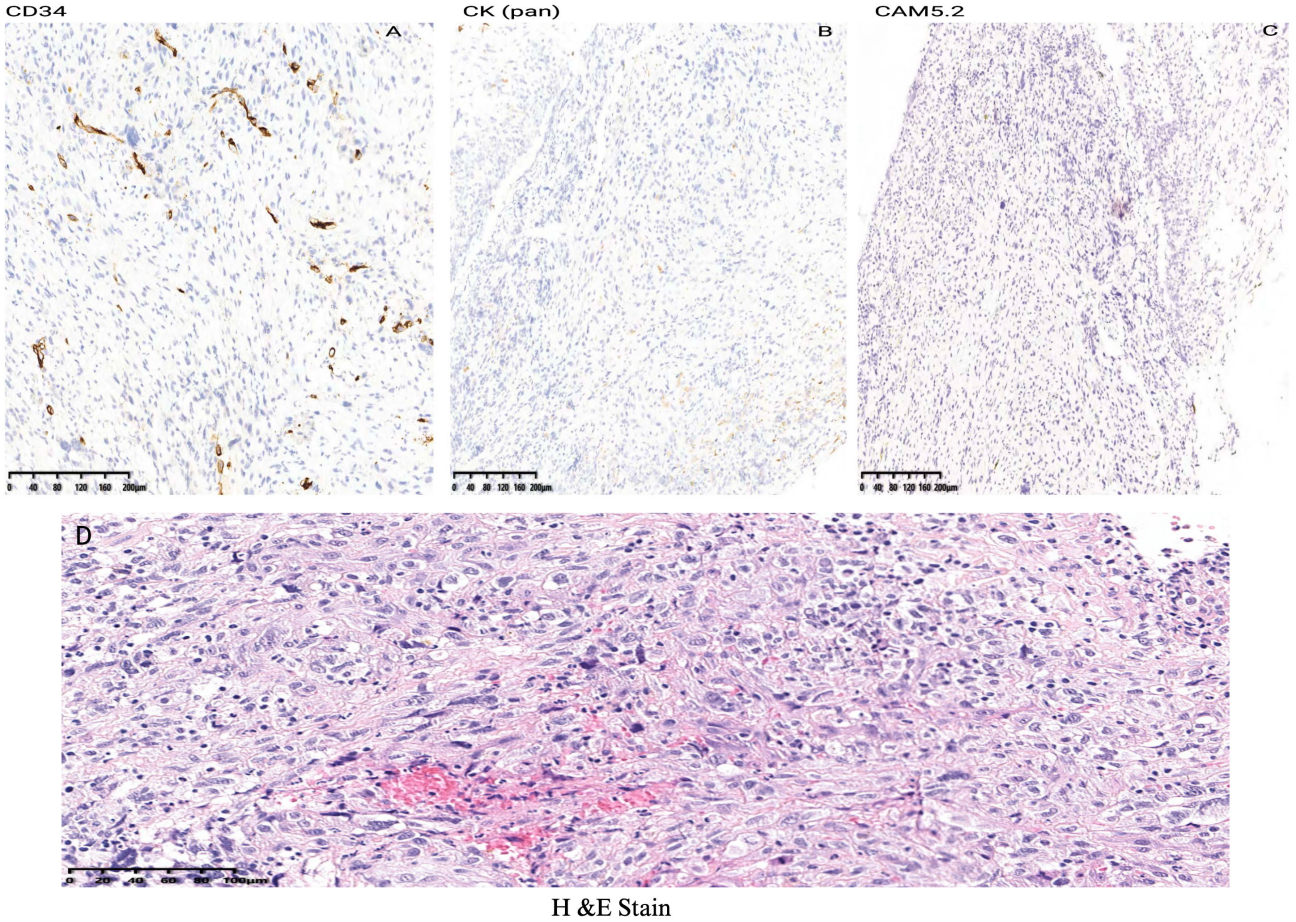
Immunohistochemical results indicated the following profiles of LSC case report: tumor cells CD34 (-) **(A)**, CK (pan) with a small amount of cells (+) **(B)**, CAM5.2 **(C)** and **(D)** H&E staining of LSC.

**Figure 4:**
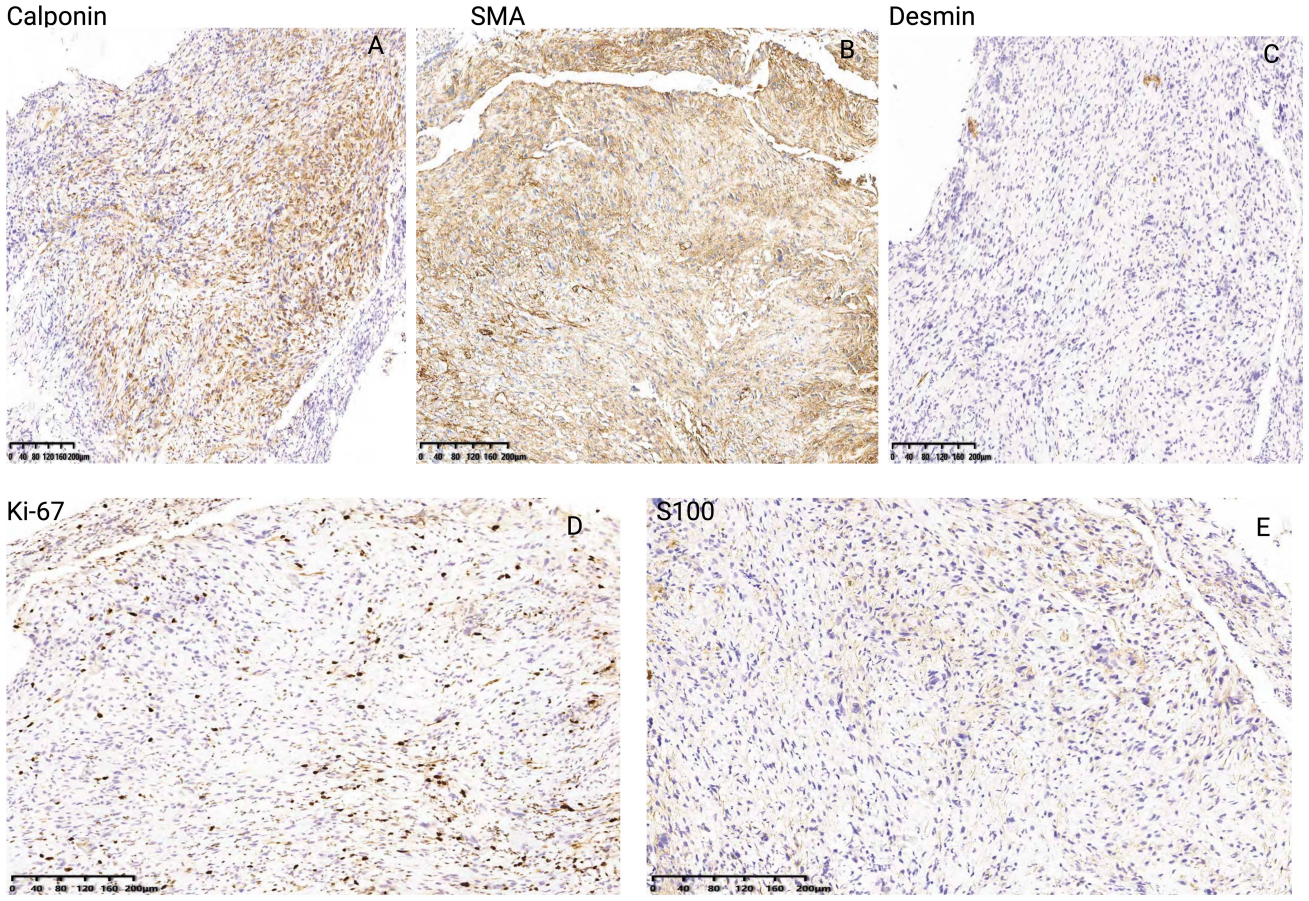
Immunohistochemical results of Calponin **(A)**, SMA **(B)**, Desmin **(C)**, Ki-67 **(D)** and S-100 **(E)** of LSC case report.

According to the tumor classification, the initial classification was given as T3N0MO. After excluding surgical contraindications, a supportive laryngoscopy biopsy was performed. The postoperative examination results revealed the presence of a malignant tumor characterized by poor differentiation and necrosis (identified in the left vocal cord lesion, biopsy). Further analysis through immunohistochemistry did not exclude the possibility of sarcomatoid carcinoma or a mesenchymal soft tissue spindle cell tumor. The immunohistochemical results indicated the following profiles: tumor cells CD34 (-) (**Figure 3A**), CK (pan) with a small amount of cells (+) (**Figure 3B**), CAM5.2 with a small amount of cells (+) (**Figure 3C**), (**Figure 3D** H&E staining of LSC), Calponin (+) (**Figure 4A**), SMA (+) (**Figure 4B**), Desmin (+) (**Figure 4C**), Ki-67 (+) 30% (**Figure 4D**) and S-100 (-) (**Figure 4E**). On March 16, 2019, a supracricoid laryngectomy combined with cricohyoidoepiglottopexy (CHEP) and left lymph node dissection (areas II, III, and IV) was performed under general anesthesia. The postoperative pathology report indicated the following findings: A malignant tumor was identified in the left ventricular zone, consistent with the prior biopsy suggesting a sarcomatoid carcinoma. The maximum tumor diameter was 1 cm, confined to the mucosal layer with adjacent striated muscle involvement; no evidence of vascular invasion or nerve infiltration was observed. During the operation, a frozen section was submitted for examination. (cut margin), left crease, right ROP, ring cartilage, epiglottis, and right arypedium were negative, and no tumor cells were found. Additionally, a malignant tumor was noted in the left sigmoid cartilage plate. The previous biopsy led to sarcomatoid carcinoma, with a maximum diameter of 0.5cm, confined to the mucosal layer, and no vasoma thrombus and nerve invasion. The left arypetis wrinkle, left ladle, right arypetis wrinkle, ring cartilage, epiglottis, and right arypep margin were negative. None of the submitted lymph nodes showed cancer metastasis: left cervical lymph node 2A: 0/1; left cervical lymph node 2B: 0/10; left cervical lymph node area 3:0/16; left cervical lymph node area 40/2. Opening closure was performed 1 year after surgery. After postoperative follow-up until January 2023, the tumor did not recur, and the patient died due to COVID-19 infection.

### Methodology

To identify potential molecular targets within LSC for exploring therapeutic opportunities, a comprehensive literature search was conducted between January 2023 and November 2024. This search utilized various scientific databases, including PubMed, Google Scholar, and Web of Science. The primary objective was to delineate the molecular pathways and specific proteins involved in the molecular signaling associated with tumor progression. To achieve this, specific keywords (Medical Subject Headings [MeSH] terms) related to “laryngeal sarcomatoid carcinoma and molecular signaling,” “laryngeal sarcomatoid carcinoma and molecular targets” and “laryngeal sarcomatoid carcinoma and therapeutic opportunities” were employed. These keywords were systematically combined using Boolean operators (AND/OR) to curate articles directly addressing laryngeal sarcomatoid carcinoma and the relevant molecular signaling proteins. The selection process commenced with a review of article titles, followed by abstracts and full texts. Duplicate articles were identified and excluded after a meticulous evaluation of the titles by each author. In total, 350 articles were considered during the selection process. Of these, 300 articles were eliminated after preliminary assessment of the titles and abstracts. Subsequently, an additional 38 articles were excluded after a thorough full-text screening. Ultimately, 12 articles met the established criteria and were deemed relevant to the topic at hand. We included only the patient tissues that were directly utilized for identifying therapeutic possibilities, excluding cell line studies.

## Results

We included a total of 12 studies related to molecular targets and their signaling pathways related to LSC aimed at exploring therapeutic possibilities and predicting them as prognostic markers for better clinical outcomes in LSC. Our observations indicate that the following molecular proteins may serve as significant targets in controlling tumor progression at various TNM stages of LSC development: TP53, CD1, Bcl2L12, P21, p27, EGFR, E-cadherin, β-catenin, FAK, NOTCH, FGFR1, PTEN, DJ-1, and TrkB. Most of the studies had male patients at higher proportions than females with smoking and alcohol consumption.

## Discussion

Sarcomatoid carcinomas have ambiguous biological implications and pathogenesis because the tumor exploration is limited to immunohistochemical, ultrastructural, and phenotype biomarkers. Nevertheless, considering the current study`s patient’s history of smoking and alcohol consumption for three decades, accompanied by symptoms of hoarseness and throat pain, the application of a traditional approach may facilitate the identification of a mass on the vocal cord. This approach would assist in tumor resection while preserving vocal function, and a biopsy could substantiate the diagnosis of Laryngeal Sarcomatoid Carcinoma (T2N0M0, Stage II). Additionally, several diagnostic challenges arise due to its rarity and histological complexity, which can lead to misdiagnosis. For instance, LSC closely resembles other spindle cell tumors, creating a diagnostic dilemma. Moreover, biopsy sampling may not capture the full spectrum of the tumor, particularly the epithelial component, which is crucial for diagnosis. This often results in misdiagnosis because these components are very small and difficult to locate. Therefore, thorough biopsies are necessary. Furthermore, histopathological examination can reveal both squamous cell carcinoma and sarcomatous components, adding to the complexity of the diagnosis. Altogether, a definitive diagnosis of LSC requires a combination of meticulous histopathological examination, judicious use of immunohistochemical markers, and careful clinical assessment correlation.

Molecular markers discussed in this study would benefit patients because they are cost-effective, rapid, and easy to implement. Pathologists may prefer these markers as surrogate indicators. For example, markers such as p53 are straightforward to assess and can identify the early stages of cancer, as well as predict therapy responses and patient outcomes. Additionally, p53 immunohistochemical (IHC) staining could aid in diagnosing predisposed tumors. Moreover, markers like E-cadherin and β-catenin levels are found to be reduced in IHC analyses of cancer samples, and this reduction correlates with the development of invasive and metastatic phenotypes. Decreased levels of E-cadherin and β-catenin in IHC are associated with tumor stages. Markers like Notch1/2/3/4 expression may serve as prognostic indicators in high-risk subgroups of cancer patients. Therefore, assessing these molecular markers would be more sensitive and specific than older methods, and, as mentioned, they are easier to use and have clinical value. However, the accuracy of these markers is open to discussion; screening for these molecular markers would offer convenience, as it only requires blood, urine, or stool samples instead of tests that involve radiation or unpleasant procedures like colonoscopy. Therefore, using these molecular markers provides an opportunity for repeated testing among the general population. Consequently, this can enhance the tests’ sensitivity and improve the chances of detecting early cancers.

### Molecular targets for improving clinical significance

Although several genes are reported to induce LSC, how these genes orchestrate the molecular signaling to change the tumor microenvironment is ambiguous. Thus, pinpointing the molecular alterations could clarify the mechanism of LSC progression and aid in identifying these molecular proteins as key therapeutic targets in this field. We included the major molecular pathways from the literature that implicates with tumorigenesis, such as epidermal growth factor receptor (EGFR), tropomyosin-related kinase B receptor (TrkB), cyclin D1, D2, D3, and NOTCH1 (**Table 1**). These molecular proteins affect the cell cycle and induce significant changes in the tumor and around the microenvironment. For instance, TP53 plays a vital role in managing genomic functions by repairing DNA damage and preventing the accumulation of harmful mutations (**Supplementary Figure 1**). A mutation in this gene bypasses this protective mechanism, resulting in tumorigenesis. In terms of LSC, a mutation in TP53 influences the apoptotic protein BCl-xl, which then affects Cyclin D1 and promotes the phosphorylation of the Retinoblastoma tumor suppressor protein (RB), facilitating cell progression in LSC (5). Research indicates that approximately 37.9% of advanced larynx cancer cases feature TP53 mutations, making it a potential prognostic and diagnostic biomarker for predicting the survival of larynx cancer patients (5). Notably, 75% of mutations occur within the DNA-binding domain, while 30% are found in the “hotspot” codons, which contributes to cancer progression (5). Studies have reported that TP53 mutations decreased the survival rate in LSC patients (5), and this may be due to the poor response to radiotherapy and increased cellular differentiation and neck LNM. Nonetheless, TP53 mutations can also render cancer cells vulnerable because they struggle to manage extensive DNA damage, leading to cell death. Further investigation is needed to determine if drugs targeting TP53 could effectively address this issue.

**Table 1:** Study characteristics of LSC patients and possible molecular targets as prognostic and diagnostic markers.

References	No of cases	Molecular target	Possible mechanism	Specific habbits	gender	TNM stages
Scheel et al., 2016 (5)	58patients	TP53	TP53 mutation affects the apoptotic protein BCl-xl, which then interferes Cyclin D1 and promotes the phosphorylation of RB for facilitating cell progression in LSC	Smoking habbits	Male (48)/ female (10)	Stage 3 (24) Stage 4 (34)
Zand et al., 2020 (6)	Out of 82, 75 were positive	CD1	Mutation in the CD1 alters the functions of RB and cellular activities such as DNA damage response checkpoint and cell cycle exit in the LSC	smoking	69 male and 13 female	(38 patients) are stage 2 and (10 patients) 4th stage
Giotakis et al., 2019 (7)	78	Bcl2L12	Cdk activity primarily inhibited by the proteins p21 and p27	Smoking	73 male/ 5 female	I (15/78) II (11/78) III (25/78) IV (27/78)
Pruneri et al., 1999 (8)	132	P21 and p27	P21 and P27 in (LSCC) can increase tumor aggression, advanced clinical stage, and metastasis via affecting Cdk activity. p21 expression correlates with elevated levels of Ki67, cyclin D, and cyclin E, while p27 is linked to accumulation of p53 and promote cell cycle progression	Smoking	129 male/ 3 (female)	Stage 1 and 2 (74) stage 3 and 4 (58)
Maurizi et al., 1996 (9)	140	EGFR	EGFR has been correlated with the anaplastic lymphoma kinase (ALK) ratio to induce neoplastic process in LSC.	–	Males (130)/ females( 10)	–
García-Cabo et al., 2020 (10)	133	E-cadherin and -catenin	E-cadherin to N-cadherin significantly influences the characteristics of epithelial-mesenchymal transitions in LSC via affecting the expression of ZEB2.	Tobacco and alcohol consumption	127 (Men) 6 (women)	Stage 1 an 2 (9), stage 3 (17) and stage 4 (33)
Aronsohn et al., 2003 (11)	35	FAK	integrin β1 and FAK signaling facilitate invasion and metastasis in LSCC. FAK phosphorylation activates the paxillin and SATA1 pathways, resulting in increased expression of MMP-2 and MMP-26, and enhances cell invasiveness and migration in LSCC.	–	34 male 1 female	–
Dai et al., 2015 (12)	55	NOTCH 1 and 2	NOTCH2 or 3 receptors can also involve in the cell growth and survival and metastasis in the LSC.			–
Monico et al., 2018 (13)	80	FGFR1	Overexpression of FGFR1 is linked to lymph node metastasis and poor survival outcomes in LSC.	–	5 female/ 69 males	–
Bruine et al., 2019 (14)	52	PTEN	PTEN is decreased in LSCC, evidenced by the increase in tumor degree, indicating in the LSC.	–	11 female and 41 male	–
Shen et al., 2011 (15)	82	DJ-1 protein	The high level of DJ-1 expression might indicate worse T stage, pTNM pathologic stage and differentiation. Survivin and DJ-1 mediated mechanism inhibits the apoptosis by mitigating the PTEN via PI3K-AKT/PKB pathways.	–	–	17 (stage 1), 16 (stage 2), 34 (stage 3), and 15 (stage 4),
Zhu et al., 2007 (16)	23	TrkB	Trk-B induce metastasis by suppressing anoikis	–	6 female and 17 male	–

Furthermore, mutation of cyclin D1, D2, D3, and Cyclin and cyclin-dependent kinase 4 and 6 (CDK4 and 6) genes are altered in the LSC, which further changes the functions of RB, altering cellular activities such as DNA damage response checkpoint and cell cycle exit (17). The upregulation of p63 is linked to the initial stages of laryngeal tumorigenesis. Bcl-2 and p53 are correlated with poor cellular differentiation, tumor progression, and lymph node metastasis (LNM), contributing to the advancement of the cancer phenotype. Pro-apoptotic isoforms like Bcl2L12 are associated with a reduced risk of patient death, whereas BCL2 and BAX do not correlate with the prognosis of LSCC patients (7). This suggests that Bcl2L12 could serve as a prognostic marker in the advanced stages of primary LSCC. CDK complexes are critical in regulating cell cycle progression, with Cdk activity primarily inhibited by the proteins p21 and p27. Notably, decreased levels of these inhibitors can impede cell cycle arrest and apoptosis, thereby facilitating cancer progression (16, 17). For example, diminished expression of p27 in LSC has been associated with increased tumor aggression, advanced clinical stage, and metastasis. Moreover, p21 expression correlates with elevated levels of Ki67, cyclin D, and cyclin E, while p27 is associated with the accumulation of p53 for promoting cell cycle progression (8). These findings suggest that p21 and p27 may be potential prognostic biomarkers for LSC. Proteins such as the EGFR have been implicated in developing LSC by modulating the epidermal growth factor/transforming growth factor α (EGF/TGFα) signaling pathway (8). Thus affecting cell transformation. Therapeutically, the level of EGFR has been correlated with the anaplastic lymphoma kinase (ALK) ratio, where EGFR overexpression is commonly observed in LSC patients presenting with poorly differentiated histological features and a low ALK ratio (8). Moreover, it has been established that EGFR, KRAS, and cyclin D1 interact synergistically to initiate neoplastic processes. Studies have reported that a decline in survival rates among LSC patients is associated with decreased levels of EGFR and cyclin D1, coupled with an increase in KRAS expression, which adversely influences prognosis (7, 8).

### Molecular targets for cancer cell invasion and metastasis in the LSCC

Metastasis is a significant contributing factor to mortality in cancer patients, defined as the spreading of cancer cells to tissues and organs distant from the original tumor site. This metastatic process primarily involves several critical steps, including invasion, intravasation, and extravasation (18). A crucial aspect of metastasis is the loss of adhesion properties in cancer cells, which facilitates their invasion into the surrounding cellular or tissue environment. Various molecules and molecular pathways play vital roles in this process, with E-cadherin and catenins being notable examples (18). E-cadherin functions as a tumor suppressor; however, its diminished expression during the epithelial-to-mesenchymal transition (EMT) can enable cancer cells to acquire metastatic capabilities. The reduction of E-cadherin expression is associated with a loss of cellular polarity and cell adhesion, fostering migratory and invasive characteristics that contribute to tumor progression (8, 18). The ratio of E-cadherin to N-cadherin significantly influences the characteristics of epithelial-mesenchymal transitions (EMTs) across various cancer types, including head and neck squamous cell carcinomas. Notably, lower expressions of E-cadherin have been observed in the LSC and are primarily associated with poor tumor differentiation and advanced T-stage (18). Research indicates that both E-cadherin and β-catenin levels are reduced in immunohistochemical (IHC) analyses of LSC samples, and this reduction correlates with the occurrence of cervical metastases (18). This phenomenon may be attributed to the expression of Zinc finger E-box-binding homeobox (ZEB2), a transcriptional repressor that initiates EMTs by downregulating E-cadherin expression, thereby enhancing tumor invasiveness (**Supplementary Figure 2**). Consequently, ZEB2 expression may serve as a prognostic biomarker in LSC, alongside E-cadherin, which acts as an EMT biomarker reflecting oncogenesis, tumor development, and metastasis of LSC. Furthermore, targeting the TGF-β/Smads pathway may also represent a valuable prognostic biomarker as it plays a critical role in activating EMT (18).

Integrins are cell surface receptors that play a significant role in the migration and invasion of cancer cells, contributing to the phenomenon of drug resistance. Notably, integrin β1 has been implicated in promoting both invasion and radioresistance in LSC (19). Evidence suggests that the overexpression of integrin β1 in LSC correlates with a poor survival rate, suggesting that integrin β1 may serve as a potential therapeutic target for this malignancy. One proposed mechanism involves the mediating effects of integrin β1 and focal adhesion kinase (FAK) signaling pathways, which facilitate invasion and metastasis in LSC (19). Additionally, the interaction of integrin β1 with CD147 has been shown to rewire metabolic reprogramming that is crucial for tumor development (19). Furthermore, selectin-dependent invasion and metastasis have been associated with cancer progression (20). The knockdown of selectins has been observed to reduce metastatic formation in LSC (20). FAK expression is also linked to laryngeal dysplasia and subsequent invasion in LSC. It appears that ECM integrins activate FAK, thereby enhancing cell survival and proliferation. The FAK-Src complex interacts with Ras-GTPase activator protein SH3 domain-binding protein 1, which inhibits the apoptosis process through the activation of various signaling pathways, including Ras/MAPK, TGF-β/Smad, and Src/FAK, as well as p53 (20). FAK affects the expression of CDK inhibitors p21 and p27, ultimately facilitating tumor progression in LSC. In the context of tumor invasion and migration, FAK knockdown has been shown to inhibit these processes by reducing the activities of matrix metalloproteinases MMP-2 and MMP-9 (21). Studies have demonstrated that FAK phosphorylation activates the paxillin and SATA1 pathways, resulting in increased expression of MMP-2 and MMP-26. Thus, it enhances cell invasiveness and migration in LSCC (22, 23).

Next, the changes in the NOTCH signaling pathway affect tumor regulation (24). For example, NOTCH1 is linked to LNM and tumor progression in LSC patients. For example, the silence of NOTCH1 in the laryngeal carcinoma Hep-2 cell line affects the migration and invasion and promotes metastasis (25). In addition, other NOTCH components like NOTCH2 or 3 receptors can also be involved in cell growth and survival, and metastasis in the LSC patients (25). Next, 5-hydroxytryptamine (serotonin) receptor 7 (HTR7) is involved in the progression of LSC via activating AKT pathway (26). For example, overexpression of HTR7 has decreased the survival rate of patients with laryngeal squamous cell cancer, suggesting that HTR7 can be an independent prognostic factor for LSC (26). Possibly, the phosphorylation of AKT by HTR7 is linked with the tumor progression. Noncoding RNAs such as miR-132 can promote laryngeal cancer proliferation and growth via targeting FOXO1, resulting in the activation of PI3K/AKT pathway (27). TRA2β is attributed to lymph node metastasis, proliferation, growth, and invasion and inhibits apoptosis in the LSC by activating PI3K/AKT (28). Fibroblast growth factor receptor 1 (*FGFR1)* plays a crucial role in the invasion, metastasis, and causing drug resistance to LSC, and FGFR1 can be an independent prognostic factor for LSC, mainly overexpression of FGFR1 is linked to lymph node metastasis and poor survival outcomes. Studies have shown that over-expression of FGFR1 is an important factor for malignant evolution and progression of laryngeal SCC (29). Another tumor suppressor gene PTEN that regulates several cellular functions such as proliferation, protein synthesis, and cell survival (29). A study has shown that PTEN is decreased in LSCC, evidenced by the increase in tumor degree, indicating that PTEN could be an important prognostic marker of LSCC tumor aggressiveness (30). PARK7 protein (DJ-1) is linked to various cancer types mainly; it influences the cancer cells transforming activity being with H-Ras/Myc, which primarily affects the S phase of the cell cycle by translocating from the cytoplasm to the nucleus (31). Studies have shown that increased levels of PARK7 in 85% of LSC patients are linked to poor survival and tumor recurrence in the LSC patients (15). A study has shown that silencing RNA targeting PARK7 significantly increased the PTEN expression, which resulted in an increase in cell death and decreased cell proliferation and invasion in the laryngeal cancer cells (32), and increase of PARK7 triggers the surviving expression, resulting in the inhibition of apoptosis and cell proliferation of laryngeal carcinoma cells (15). Tropomyosin-related kinase B receptor (TrkB) plays various roles in inducing tumor progression, such as increasing invasion, metastasis, and angiogenesis and inducing resistance against cancer treatments (32). TrkB overexpression is linked to metastatic laryngeal cancer cell lines, and it drives EMT by regulating c-Src-mediated activation of PI3K/AKT signal pathway, suggesting the therapeutical opportunity of TrkB to counteract metastasis in the LSC (33).

### Genetic/molecular alterations involved in sarcomatoid transformation

Sarcomatoid carcinoma is a rare morphological variant with distinctive histological features. It can exist as either a sarcomatoid form or a typical squamous phenotype. Despite its aggressive behavior, poor survival rates, and higher levels of tumor programmed death-ligand 1 (PD-L1), the mechanisms behind its evolution and progression remain unknown. However, EMT is a widely accepted theory, with contrasting hypotheses regarding the development of these tumors as follows: a monoclonal origin from an undifferentiated stem cell that generates both mesenchymal and squamous components, and sarcomatoid carcinoma is a multiclonal origin where these components arise independently from different cell types. However, studies reported that these types of carcinomas from different cell types showed similar molecular and genetic features, supporting the monoclonal hypothesis, and the differentiation and morphogenesis of these carcinomas are organized by coordinated genetics and molecular events of both epithelial and mesenchymal elements, which can provide crucial information of sarcomatoid transformation. Although the underlying molecular events are unknown, the loss of heterozygosity in chromosome 17p, and subsequent molecular progression is responsible for sarcomatoid transformation (34). Tumor growth factor-β (TGF-β), epithelial growth factor (EGF), and insulin-like growth factor (IGF) have been linked to sarcomatoid transformation (35). For example, TGF-β promotes the EMT via MAPK through Hic-5, a focal adhesion protein that is crucial for maintaining the mesenchymal phenotype, accompanied by RhoA activation (36). Additionally, higher expression of Src is associated with sarcomatoid transformation. The activation of these molecular events alters mesenchymal morphology, increasing the motility and invasiveness of tumors. Mutations in the pathways of VENTX, HIF-1α, and SUMOylation induce DNA damage and drive proliferation toward EMT (37). For instance, sentrin/SUMO2/3-specific protease (SENP3) modifies the removal of SUMO2/3, which leads to increased cell proliferation, tumorigenesis, and EMT through STAT3 activation (38). Y-box binding protein 1 (YB-1) is a conserved protein that induces epithelial-to-mesenchymal transition and further metastasis by binding to HIF-1α and triggering the translation of HIF1A messages, enhancing metastatic capacity in sarcomatoid carcinoma (39). Moreover, other new mutations in sarcomatoid carcinoma, such as integrin cell surface interactions, WNT, MAPK, and BRAF signaling pathways, induce EMT phenotypes (37). For example, mutations in WNT drive the proliferation of mesenchymal stem cells through the TCF/β-catenin target gene CDC25A, which is crucial for cell cycle progression (40). Targeting these molecular proteins may offer viable clinical strategies.

## Conclusion

Laryngeal sarcomatoid carcinoma is a rare type of cancer, and its clinical manifestations and imaging manifestations are not significantly specific, and it is confirmed only based on pathological examination. Treatment options are still controversial. The particular treatment regimen still depends on the specific condition of the patient. In this case, CHEP + left lymph node dissection was performed without chemoradiation, with no postoperative recurrence or metastasis. Regarding molecular proteins as prognostic and diagnostic markers in the LSC, we found TP53, CD1, Bcl2L12, P21, p27, EGFR, E-cadherin, β-catenin, FAK, NOTCH, FGFR1, PTEN, DJ-1, and TrkB are the possible markers to inhibit the tumor stages as they are involved in the cell cycle progression and cell cycle arrest in the LSC. However, further research is warranted on these molecular markers to elucidate their dual nature, particularly their potential role in inducing DNA damage in cancer cells.

## Data Availability

The raw data supporting the conclusions of this article will be made available by the authors, without undue reservation.

## Supplementary material

Supplementary Figure 1 Molecular signaling pathways for improving clinical significance in LSC. P53 and p63 mutations induce LSC metastasis by affecting Bcl-xl, Ki67, Cyclin D1, D2, and cyclin E and Retinoblastoma (RB) phosphorylation.

Supplementary Figure 2 Potential Prognostic Biomarkers for Laryngeal sarcomatoid carcinoma (LSC). The blue arrows indicate the molecular signaling pathways involved in the induction of tumorigenesis, impacting apoptosis, cell proliferation, differentiation, and metastasis.
