# Supplementary figures and images for: Laryngeal sarcomatoid carcinoma: a case report and literature review on potential molecular targets for therapeutic opportunities
Source: Front Oncol. 2025 Mar 31;15:1549790. doi: 10.3389/fonc.2025.1549790 (PMC11994598; doi:10.3389/fonc.2025.1549790)

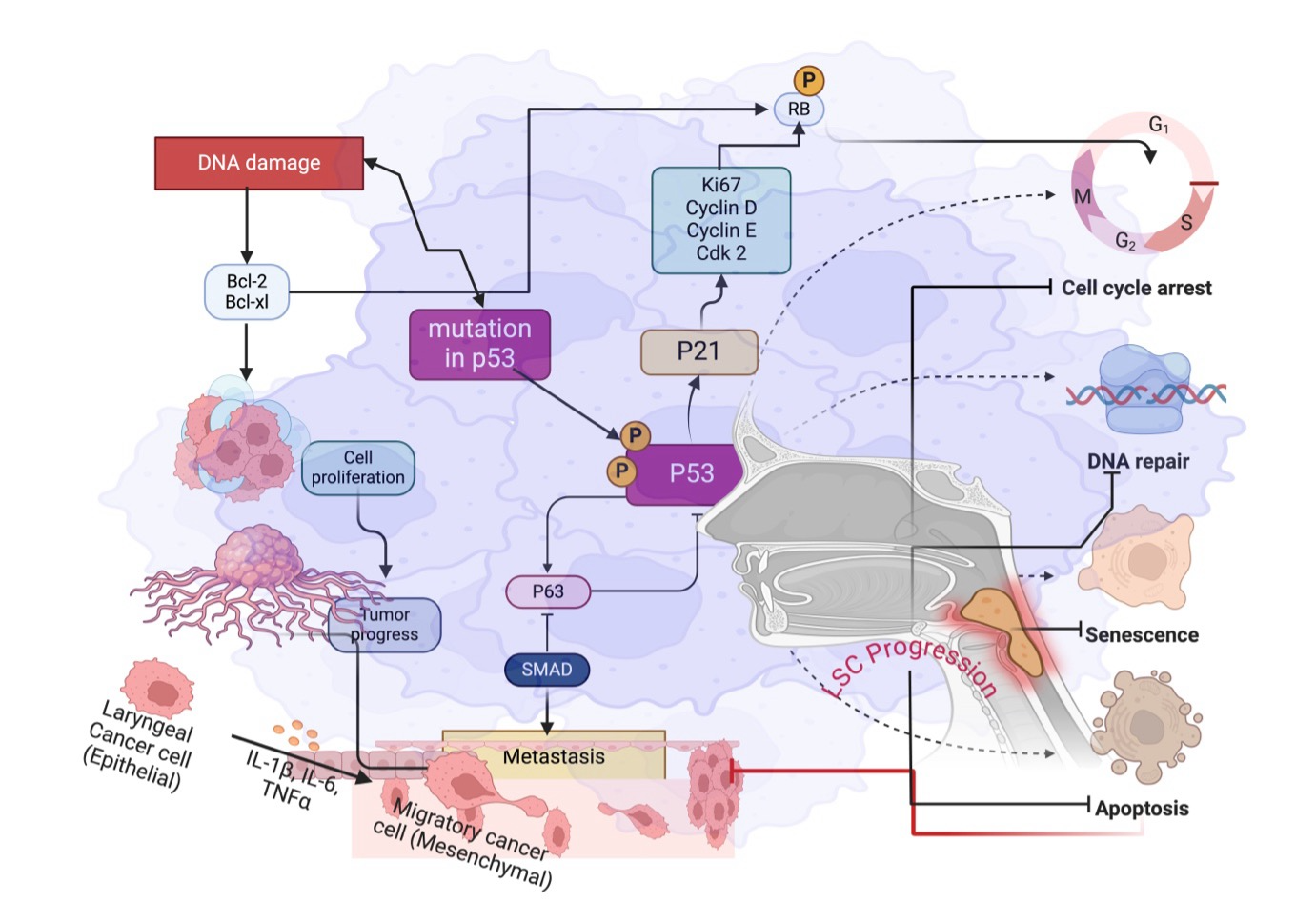

Supplement: Supplementary Figure 1 — Molecular signaling pathways for improving clinical significance in LSC. P53 and p63 mutations induce LSC metastasis by affecting Bcl-xl, Ki67, Cyclin D1, D2, and cyclin E and Retinoblastoma (RB) phosphorylation. [file Image1.tiff]

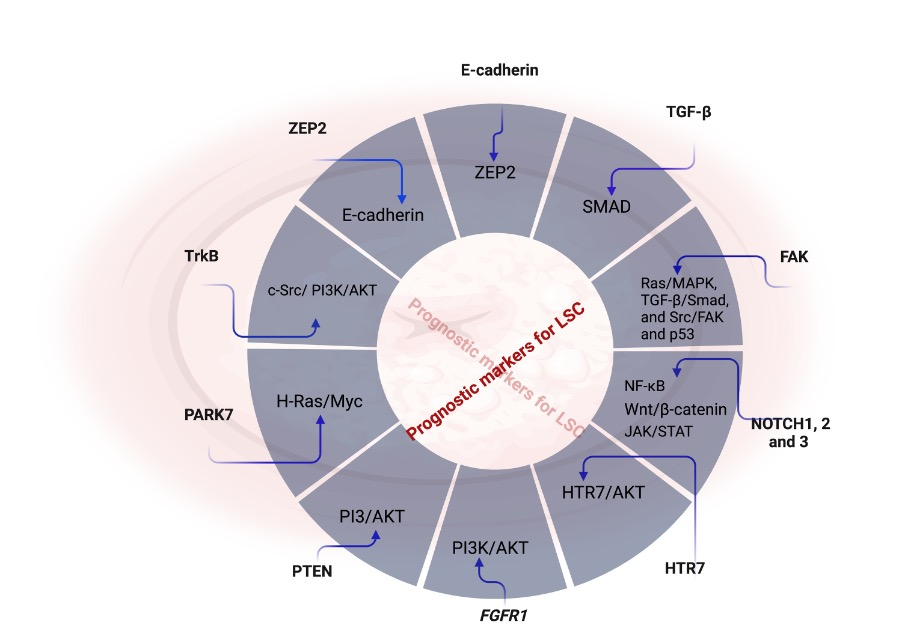

Supplement: Supplementary Figure 2 — Potential Prognostic Biomarkers for Laryngeal sarcomatoid carcinoma (LSC). The blue arrows indicate the molecular signaling pathways involved in the induction of tumorigenesis, impacting apoptosis, cell proliferation, differentiation, and metastasis. [file Image2.tiff]
